# Contributions from specific and general factors to unique deficits: two cases of mathematics learning difficulties

**DOI:** 10.3389/fpsyg.2014.00102

**Published:** 2014-02-13

**Authors:** Vitor G. Haase, Annelise Júlio-Costa, Júlia B. Lopes-Silva, Isabella Starling-Alves, Andressa M. Antunes, Pedro Pinheiro-Chagas, Guilherme Wood

**Affiliations:** ^1^Developmental Neuropsychology Laboratory, Department of Psychology, Universidade Federal de Minas GeraisBelo Horizonte, Brazil; ^2^Programa de Pós-graduação em Saúde da Criança e do Adolescente, Faculdade de Medicina, Universidade Federal de Minas GeraisBelo Horizonte, Brazil; ^3^INSERM, U992, Cognitive Neuroimaging UnitGif sur Yvette, France; ^4^CEA, DSV/I2BM, NeuroSpin CenterGif sur Yvette, France; ^5^Department of Neuropsychology, Institute of Psychology, Karl-Franzens-University of GrazGraz, Austria

**Keywords:** endophenotype, mathematics learning difficulties, number sense, verbal numerical representations, phonological processing, dyslexia

## Abstract

Mathematics learning difficulties are a highly comorbid and heterogeneous set of disorders linked to several dissociable mechanisms and endophenotypes. Two of these endophenotypes consist of primary deficits in number sense and verbal numerical representations. However, currently acknowledged endophenotypes are underspecified regarding the role of automatic vs. controlled information processing, and their description should be complemented. Two children with specific deficits in number sense and verbal numerical representations and normal or above-normal intelligence and preserved visuospatial cognition illustrate this point. Child H.V. exhibited deficits in number sense and fact retrieval. Child G.A. presented severe deficits in orally presented problems and transcoding tasks. A partial confirmation of the two endophenotypes that relate to the number sense and verbal processing was obtained, but a much more clear differentiation between the deficits presented by H.V. and G.A. can be reached by looking at differential impairments in modes of processing. H.V. is notably competent in the use of controlled processing but has problems with more automatic processes, such as nonsymbolic magnitude processing, speeded counting and fact retrieval. In contrast, G.A. can retrieve facts and process nonsymbolic magnitudes but exhibits severe impairment in recruiting executive functions and the concentration that is necessary to accomplish transcoding tasks and word problem solving. These results indicate that typical endophenotypes might be insufficient to describe accurately the deficits that are observed in children with mathematics learning abilities. However, by incorporating domain-specificity and modes of processing into the assessment of the endophenotypes, individual deficit profiles can be much more accurately described. This process calls for further specification of the endophenotypes in mathematics learning difficulties.

## Introduction

The cognitive underpinnings of arithmetic are highly complex (Rubinsten and Henik, 2009). One proposal is that arithmetic requires three types of symbolic and nonsymbolic number representations (Dehaene, 1992). The most basic form of numerical representation is nonsymbolic, analogic and approximate and corresponds to the number sense or the ability to discriminate numerosities. This ability can be described by Weber–Fechner's law, which measures the precision of the internal representation of numbers (Moyer and Landauer, 1967; Izard and Dehaene, 2008; Piazza, 2010). Precise numerical magnitude representations are related to phonologically and orthographically coded verbal numerals and visually based Arabic numerals (Dehaene and Cohen, 1995).

The number sense acuity is predictive of math achievement in both typical (Halberda et al., 2008; Mazzocco et al., 2011a) and disabled individuals (Piazza et al., 2010; Mazzocco et al., 2011b). Moreover, general cognitive resources are also involved in number processing, and calculations involve visuospatial abilities (Venneri et al., 2003), finger gnosias (Costa et al., 2011), phonological processing (De Smedt and Boets, 2010; De Smedt et al., 2010), working memory and executive functions (Camos, 2008; Pixner et al., 2011; Zheng et al., 2011).

The phenotypic presentation of mathematics learning disability and developmental dyscalculia (DD) is heterogeneous and includes a combination of the cognitive mechanisms that underlie arithmetic (Geary, 1993; Wilson and Dehaene, 2007). Because there are no consensual cognitive or biological markers, DD is operationally defined as persistent and severe difficulties in learning math in children of normal intelligence, that cannot be attributed to neurosensory impairment, sociodemographic, and emotional factors, or lack of adequate educational experiences (American Psychiatric Association, 2000; World Health Organization, 2011). The nosological complexity of DD is compounded by its frequent comorbidity with other disorders, such as dyslexia (Landerl and Moll, 2010) and attention-deficit-hyperactivity disorder (ADHD, Gross-Tsur et al., 1996). Comorbidity can be explained by chance co-occurrences or by shared underlying mechanisms. The present evidence is still insufficient to decide about the role of comorbidity in characterizing DD (Rubinsten and Henik, 2009).

One possible way to solve the conundrum of DD's nosological validity is to consistently characterize implicated cognitive mechanisms as endophenotypes, in other words, as intermediate constructs between the interacting environmental and genetic etiologies and the phenotypic expression (Bishop and Rutter, 2009). A reliable endophenotype of number sense impairment has been gradually emerging (Piazza et al., 2010; Mazzocco et al., 2011b). However, restricting the definition of DD to individuals with more basic number processing impairments related to a number sense or number module (Reigosa-Crespo et al., 2012) would exclude from the domain of coverture of DD children and adolescents whose math learning difficulties could be persistent and of varying degrees of severity but associated with other cognitive mechanisms, such as phonological processing disorders (De Smedt and Boets, 2010).

Moreover, cognitive mechanisms that underlie math achievement and are potentially implicated in math learning difficulties could be classified as domain-specific or domain-general (Butterworth and Reigosa, 2007). Math-specific cognitive mechanisms include number sense (e.g., symbolic and nonsymbolic number comparison and estimation, number line estimation) and knowledge of the number system (Cowan and Powell, 2013). Domain-general mechanisms associated with math achievement and underachievement include phonological processing (Hecht et al., 2001), intelligence, processing speed, working memory, and executive functions (Cowan and Powell, 2013). It is increasingly recognized that DD can thus be characterized as primary, associated with number sense deficits, or secondary, associated with domain-general factors (Price and Ansari, 2013, for similar conceptions, see also Rubinsten and Henik, 2009; Reigosa-Crespo et al., 2012).

We argue that, in addition to being influenced by primary and secondary cognitive factors, the achievement profile of kids who struggle to learn math could also be affected by the nature of the information processing strategy that is deployed. An important research tradition in cognitive psychology, which dates back at least to Shiffrin and Schneider (1977), distinguishes between automatic (data-driven, bottom–up, effortless) and controlled (concept-driven, top–down, effortful) processing (Hasher and Zacks, 1979; Logan, 1988; Birnboim, 2003).

Evidence is still accumulating and is often inconsistent, but there are data that support impairments of both automatic and controlled processing in math learning difficulties. Impairments in the rapid automatized naming (RAN) of numbers (Bull and Johnston, 1997), a lack of the congruency effect in the number-size interference task (Rubinsten and Henik, 2005), and impairment in symbolic (with sparing of nonsymbolic) number comparisons (Rousselle and Noël, 2007) have been interpreted as evidence for an automatization deficit in DD. Impairments of several subcomponents of the central executive in DD have often been described (Bull and Scerif, 2001; van der Sluis et al., 2004; Geary et al., 2007; Raghubar et al., 2010, see also Kaufmann et al., 2004; de Visscher and Noël, 2013). This literature indicates that math achievement could be associated with both domain-specific and domain-general cognitive factors. Moreover, these two dimensions could interact with different modes or strategies of information processing according to the nature of the task.

In general, it is possible to say that researchers agree as to the cognitive factors that are implicated in math learning difficulties. Disagreement arises when the relative importance of each factor or their possible interactions or lack of interaction are considered. One possibility is a multiple-deficit model, according to which math learning difficulties are the epigenetic outcome of multiple interacting mechanisms (Cowan and Powell, 2013). Another possibility is that different types of DD are explained by impairments in different non-interacting endophenotypes. One of the most important endophenotypes that is implicated in dyscalculia is a number sense or a number module deficit (Reigosa-Crespo et al., 2012). Single-case studies of individuals with math learning difficulties could constitute an opportunity to test these concurrent models of cognitive impairments in dyscalculia.

Although not without its critics (Thomas and Karmiloff-Smith, 2002), the logic of double-dissociation in cognitive neuropsychology has also been applied in the context of developmental disorders, to more specifically characterize the endophenotypes that are implicated (Temple, 1997; Temple and Clahsen, 2002; White et al., 2006a,b; de Jong et al., 2006, 2009). In cognitive neuropsychology, it is generally assumed that if two cognitive processes double-dissociate or present complementary patterns of spared and impaired functions in two different patients, then this pattern is an indication of different underlying neural substrates (Temple, 1997).

A possible double-dissociation in the field of learning disabilities is the case of the underlying cognitive mechanisms of DD and dyslexia. Evidence indicates that children with DD could be selectively impaired in number sense tasks, while dyslexia impairs phonological processing (Rubinsten and Henik, 2006; Landerl et al., 2009). Analysis has been performed on a series of single-case-generated evidence that is compatible with this interpretation (Tressoldi et al., 2007). The sole occurrence of DD and the sole occurrence of dyslexia, when associated with different cognitive profiles, suggest that these two disorders constitute distinct entities. At least in certain cases, the co-occurrence of DD and dyslexia could represent a true comorbidity, without a shared etiopathogenic variance (Landerl and Moll, 2010).

Double-dissociation logic has also been used to refine the phenotype of DD, characterizing subtypes that are related to impairments in specific cognitive components. A double dissociation has been observed in Arabic number processing. A case described by Temple (1989) presented a specific difficulty in reading Arabic numbers. The opposite difficulty of writing Arabic numbers was found by Sullivan et al. (1996). Similar to what is observed in adults with acquired acalculia, Temple (1991) demonstrated the existence of a double dissociation between procedural calculation impairment and a fact retrieval deficit. Specific fact retrieval deficits were later corroborated by Temple and Sherwood (2007) in a group study. Two additional single-case studies described specific impairments in math facts retrieval, uncovering a role for executive function and automatization in the deficits (Kaufmann, 2002; Kaufmann et al., 2004; de Visscher and Noël, 2013). Moreover, more complex interactions between magnitude processing and procedural knowledge also can be observed in the carry over operation when solving addition problems (Klein et al., 2010). A number sense deficit impairing cardinality and sparing ordinality was observed in an earlier case described by Ta'ir et al. (1997).

This line of reasoning suggests, then, that single-case studies that use double-dissociation logic could play an important role in clearing the complexity that underlies phenotypic manifestations of DD and in establishing the relevant endophenotypes. Investigations on the number sense endophenotype using contemporary experimental measures are missing in the single-case literature. In this study, the aim is to contrast the patterns of cognitive deficits in two children at approximately 10 years of age with persistent math learning difficulties that are associated with distinct cognitive profiles. H.V., a 9-year-old girl, has math learning difficulties in the context of number sense inaccuracy, while G.A, a 10-year-old boy, presents math difficulties that are associated with developmental dyslexia and a phonological processing disorder. Neither of the children fulfilled the criteria for a more severe math learning disorder or disability. Instead, they were classified as having math learning difficulties, in other words, a performance below the 25th percentile on a standardized achievement test (Mazzocco, 2007). Performance on the Arithmetic subtest of the WISC-III was also not impaired in either of the children. Notwithstanding spared psychometric performance on achievement and intelligence tests, these two children presented persistent difficulties in specific domains of arithmetic, which were severe enough to cause low grades and to justify clinical referral.

The two cases were considered for analysis because of the comparable ages, similar sociodemographic backgrounds, normal or above average intelligence and impairment patterns that were suggestive of specific deficits in math learning difficulties. Standard neuropsychological assessment revealed specific impairments in the number sense in H.V. and in phonological processing in G.A. A more detailed assessment followed these observations.

Both domain-general and domain-specific cognitive mechanisms were included in the assessment (Butterworth and Reigosa, 2007; Cowan and Powell, 2013). Specific math assessment was based on two widely used cognitive models (McCloskey et al., 1985; Dehaene and Cohen, 1995). In the numerical domain, the following assessments were performed: numerical transcoding, calculation, simple word problems and the approximate number system (ANS).

Selection of domain-general assessments included the following functions: general intelligence (Deary et al., 2007), working memory (Geary et al., 2007; Raghubar et al., 2010), and executive functions (van der Sluis et al., 2004). Moreover, we used both non-numerical (Victoria Stroop, Strauss et al., 2006) and numerical stimuli (Five-digits Test, Sedó, 2007) when testing executive functions and interference (see the rationale in Raghubar et al., 2010). Some aspects of our assessment protocol deserve further discussion. Phonological processing has been implicated in math learning (Hecht et al., 2001), mostly in the context of developmental dyslexia. A specific subtype of verbal dyscalculia has even been proposed (Wilson and Dehaene, 2007). Notwithstanding its theoretical plausibility, there is scarce evidence for a visuospatial subtype of dyscalculia (Geary, 1993; Wilson and Dehaene, 2007). Impairment of more executive aspects of visuospatial processing in math achievement has been reported, mostly in the context of the so-called nonverbal learning disability (Venneri et al., 2003). Wilson and Dehaene (2007) consider the possibility that impairments in the ANS and deficits in visuospatial attention could constitute two different subtypes of dyscalculia. It is important then to assess visuospatial and visuo-constructional abilities to check for the possibility of a nonverbal learning disability (Venneri et al., 2003; Fine et al., 2013). Finally, assessment of finger gnosias and motor dexterity were obtained because of their association with math learning difficulties (Costa et al., 2011; Lonnemann et al., 2011). Finger gnosias can underlie finger counting, which is an important offloading mechanism that liberates working memory resources at the beginning of formal math learning (Costa et al., 2011). Motor impairment could provide clues regarding the presence of minor brain insult (Denckla, 1997, 2003; Batstra et al., 2003).

## Methods

Considering the hypothesis that modes of information processing interact with the domain-specificity of stimuli in the genesis of learning difficulties, we employed tasks assessing automatic and controlled processing in both general and math-specific domains. General automatic processing was assessed using RAN of colors in the Victoria Stroop test. Numerical automatic processing was assessed by means of RAN of digits and speeded counting in the Five-digits Tests, nonsymbolic and symbolic number comparison tasks and by retrieval of arithmetic facts. Domain-general controlled processing was tapped by backward Corsi blocks span and the color-word interference phase of the Victoria Stroop test. Controlled processing in the numerical domain was evaluated with the backward Digit span and Inhibtion and Switching tasks of the Five-digits Test, as well as by word problems and working memory-dependent items in the numerical transcoding tasks. A simple reaction time task and the Nine-hole Peg Test were used to control, respectively for more basic aspects of alertness and motoric function.

### Case reports

H.V. and G.A. were selected from cases at an outpatient facility for mathematical learning disabilities in Belo Horizonte, Brazil. Parents gave their written informed consent. In addition, informed consent was orally obtained from the children. Anamnestic information was obtained from the mothers of the two children.

#### H.V.

H.V. is a well-adjusted girl from a middle-class and supportive family, attending the third grade at a private school. She had just completed 9 years of age by the time of evaluation. H.V. had difficulties in telling time on analogic and digital displays and estimating/comparing object sets (e.g., telling if a bookshelf had more or fewer books than another). She struggled to learn the math facts, to understand the place-value system and to solve word math problems. She uses fingers as a support to perform even the most simple additions and subtractions. Her learning difficulties are highly specific to math because her intelligence and achievement in other domains are above the average expected for her age. No major developmental problems were reported.

#### G.A.

G.A. is a well-adjusted boy from a middle-class and supportive family, who was 10 years and 2 months at the time of the neuropsychological assessment. He was attending the third grade at a public school. During his infancy, G.A. was submitted to several ear canal draining procedures that were related to recurrent otitis media. After the last surgery, his hearing and speech improved. His hearing is now normal and he was re-evaluated by a speech therapist who confirmed he has already improved from his previous difficulties. However, occasionally, he still mispronounces some of the more complex words, those that are less frequent and multi-syllable words that have consonantal clusters. G.A. was referred due to early and persistent difficulties with reading/spelling and math. His reading/spelling difficulties are severe. His math difficulties are milder but are also persistent and are mostly related to word problem solving. Clinically, G.A. presents difficulties with attention. A tentative diagnosis of ADHD was made by another clinician.

### Procedures

First, a general neuropsychological assessment was conducted, and the performances of both H.V. and G.A. were compared to available published norms. Table 1 lists the neuropsychological tests and their sources. Afterward, an experimental study was conducted to specifically investigate math cognition in both cases. In the experimental investigation, the performances of H.V and G.A. were compared to two control groups that were individually matched by gender, educational level, age, and socioeconomic status. In Brazil, the type of school is an important indicator of socioeconomic status because private schools generally offer better instruction than public schools (Oliveira-Ferreira et al., 2012). For this reason and because of the age differences between the two patients, separate control groups were used for the comparisons. The controls were selected among the participants of a population-based research project on math learning difficulties that was approved by the local ethics review board. Parents gave written informed consent, and the children gave their oral consent.

**Table 1:** **Neuropsychological instruments**.

**Domain**	**Test**	**References**
Psychosocial functioning	CBCL—Child Behavior Checklist responded to by parents	Achenbach et al., 2008; Rocha et al., 2012
School achievement	TDE—Teste de Desempenho Escolar (School Achievement Test)	Stein, 1994; Oliveira-Ferreira et al., 2012
Intelligence	Raven's colored progressive matrices	Angelini et al., 1999
	Wechsler intelligence scale for children 3° ed.	Figueiredo, 2002
Motor dexterity	9-HPT: Nine-hole peg test	Poole et al., 2005
Visuospatial abilities	Copy of the Rey–Osterrieth complex figure	Oliveira, 1999
Short-term and working memory	Corsi blocks	Santos et al., 2005
	WISC-III digits	Figueiredo and do Nascimento, 2007
	Auditory consonantal trigrams	Vaz et al., 2010
Phonological processing	Phoneme elision task	Lopes-Silva et al., 2014
	Pseudoword repetition	Santos and Bueno, 2003
	Pseudoword reading	Same stimuli as in Santos and Bueno (2003)
Executive functions	Color-word interference in the Victoria stroop	Charchat-Fichman and Oliveira, 2009
	Five-digits test	Sedó, 2004

The test performance of both cases was compared either to normed values, in the general neuropsychological assessment, or to the reference given by their individually selected control groups, in the math-cognitive assessment. Different statistical procedures that were based on psychometric single-case analysis (Huber, 1973; Willmes, 1985), one person vs. small sample comparisons (Crawford et al., 2010) and criterion-oriented methods (Willmes, 2003), were employed in these comparisons.

H.V.'s performance was compared to that of a group of 8 girls [mean age = 113 (*SD* = 3) months] from 3rd grade of a private school in Belo Horizonte, Brazil. All of them had intelligence performance that was well above the mean (percentile ranks in the Raven's Colored Progressive Matrices ranged from 70 to 95) and no learning difficulties. G.A.'s performance, in turn, was compared to that of 17 boys [mean age = 117 (*SD* = 4) months] from the 3rd grade of two public schools in Belo Horizonte, Brazil. The percentile ranks in the Raven' Colored Progressive Matrices of this control group ranged from 50 to 99, which was comparable to that of G.A.'s.

### Instruments

In the following section, the more specific cognitive tests and tasks will be described in greater detail.

#### Brazilian school achievement test (TDE; Stein, 1994)

The TDE is a standardized test of school achievement (Oliveira-Ferreira et al., 2012) and comprises arithmetic, single-word spelling, and single-word reading. Specific norms are provided for school-age children between the second and seventh grade. Reliability coefficients (Cronbach α) of TDE subtests are 0.87 or higher. Children are instructed to work on the problems to the best of their capacity but without time limits.

#### Nine-hole peg test (9-HPT, Poole et al., 2005)

The 9-HPT is a timed test in which nine pegs should be inserted and removed from nine holes in the pegboard with the dominant and non-dominant hand. The pegboard is placed horizontally in front of the child, in such a way that the compartment that contains the pegs is on the side of the hand to be tested, while the compartment with the holes is on the contralateral side. Children must pick up one peg at a time. The test is performed two times with each hand, with two consecutive attempts with the dominant hand followed immediately by two consecutive attempts with the non-dominant hand. The scores were calculated based on the mean time for each hand.

#### Handedness ascertainment

Lateral preference was investigated by means of tasks that examine the ocular, hand, and foot preference based on Lefèvre and Diament (1982). The child was instructed to look through a hole, to kick and to throw a ball, three times each. The result was given by the side the child had chosen most of the time.

#### Right–left orientation test

This test is based on Dellatolas et al. (1998). It has 12 items of right and left body part recognition that involves simple commands regarding the child's own body, double commands (direct and crossed) toward the child's body, and pointing commands to single lateral body parts of an opposite-facing person. The score system is based on the number of correctly pointed parts of the body. Internal consistency was assessed with the Kuder–Richardson reliability coefficient, which was high (KR-20 = 0.80) (Costa et al., 2011).

#### Finger localization task

This 24-item task was also based on Dellatolas et al. (1998), and it was used to assess finger gnosia. It consists of three parts: (1) localization of single fingers touched by the examiner with the hand visible (two trials on each hand); (2) localization of single fingers touched by the examiner with the hand hidden from view (four trials on each hand); and (3) localization of pairs of fingers simultaneously touched by the examiner with the hand hidden from view (six trials on each hand). A total score (that ranged from 0 to 12) was calculated for each child as well as the total score, which was the sum of the total from both hands. The internal consistency of this task is high (KR-20 = 0.79) (Costa et al., 2011).

#### Phoneme elision task

This test is a widely accepted measure of phonemic awareness (Wagner and Torgesen, 1987; Castles and Coltheart, 2004; Hulme et al., 2012; Melby-Lervåg et al., 2012). The child listens to a word and is expected to say how it would be if a specified phoneme were deleted. (e.g., “*filha*” without /f/ is “*ilha*” in English it would be “cup” without /k/ is “up”). The test comprises 28 items: in 8 of them, the child must delete a vowel, and in the other 20, a consonant. The consonants to be suppressed varied according to the place and manner of articulation. The phoneme to be suppressed could be in different positions of the words, which ranged from 2 to 3 syllables. The internal consistency of the task is 0.92 (KR-20 formula) (Lopes-Silva et al., 2014).

#### Victoria stroop task (Charchat-Fichman and Oliveira, 2009)

The Victoria Stroop task is a measure of executive function (Strauss et al., 2006). The subject is presented with three cards, each containing six rows of four items. In the first card (color), the task is to name quickly the color of 24 rectangles, which can be green, yellow, blue, or red. In the second card (word), the task is to name the colors of common words printed in green, yellow, blue, or red, ignoring their verbal content. On the third card, the stimuli are color names that are printed in an incongruent color that is never the same color as the word that is printed. The task is to name the color in which the word is printed (e.g., when the word “blue” is printed in red, the subject must say “red”). For each of the three conditions, the time to complete the naming of all of the stimuli was recorded. Additionally, the interference score (Stroop-Effect) was calculated as the quotient between the time score for the incongruent (third card) and the color (first card) conditions.

#### Five digits test

The Five Digits Test was validated and standardized in Spanish and English by Sedó (2004, 2007) as a measure of speeded counting, Arabic number reading, and inhibition and set shifting. Similar sets of stimuli are used across tasks. Automatic processing is assessed through speeded tasks of counting randomly presented star sets (up to five) and reading Arabic digits (up to five). Controlled processing is assessed through inhibition and set-shifting tasks. In the inhibition task (choosing), the child must count the number of Arabic digits instead of reading them. In the set-shifting condition (switching), the child switches from counting the number of Arabic digits in most trials to reading them when a frame surrounds the stimulus set.

The numeric and arithmetic tasks for the experimental study have been employed in previous investigations (Costa et al., 2011; Ferreira et al., 2012; Júlio-Costa et al., 2013) and are described below.

#### Simple reaction time

The computerized simple RT task is a visual detection task that is used to control possible differences in the basic processing speed that is not related to numerical tasks. In this task, a picture of a wolf (height 9.31 cm; length = 11.59 cm) is displayed in the center of a black screen for a maximum time of 3000 ms. The participant is instructed to press the spacebar on the keyboard as fast as possible when the wolf appears. Each trial was terminated with the first key press. The task has 30 experimental trials, with an intertrial interval that varies between 2000, 3500, 5000, 6500, and 8000 ms.

#### Nonsymbolic magnitude comparison task

**Figure 1:**
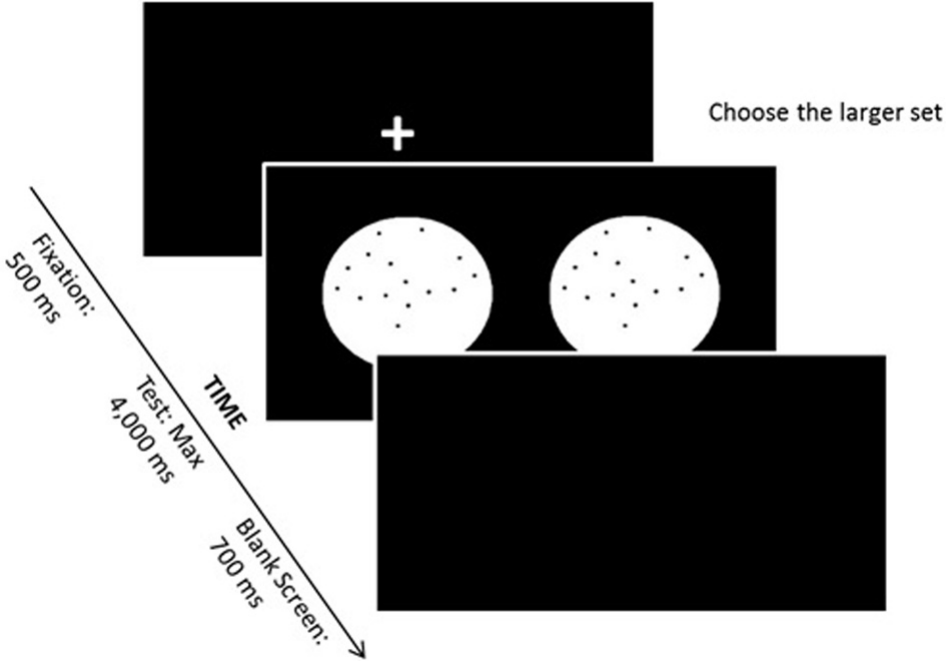
**Nonsymbolic magnitude comparison task**.

In the nonsymbolic magnitude comparison task, the participant was instructed to compare two simultaneously presented sets of dots and to indicate which set contained the larger number (see Figure 1). Black dots were presented on a white circle over a black background. On each trial, one of the two white circles contained 32 dots (reference numerosity), and the other circle contained 20, 23, 26, 29, 35, 38, 41, or 44 dots. Each magnitude of dot sets was presented 8 times. The task comprised 8 learning trials and 64 experimental trials. Perceptual variables were randomly varied such that in half of the trials, the individual dot size was held constant, while in the other half, the size of the area occupied by the dots was held constant (see exact procedure descriptions in Dehaene et al., 2005). The maximum stimulus presentation time was 4000 ms, and the intertrial interval was 700 ms. Between each trial, a fixation point appeared on the screen—a cross, printed in white, with 30 mm in each line. If the child judged that the right circle presented more dots, then a predefined key localized on the right side of the keyboard should be pressed with the right hand. In contrast, if the child judged that the left circle contained more dots, than a predefined key on the left side had to be pressed with the left hand.

#### Symbolic magnitude comparison task

In the symbolic magnitude comparison task, Arabic digits from 1 to 9 were presented on the computer screen (height = 2.12 cm; length = 2.12 cm). The visual angle of the stimuli was 2.43° in both the vertical and horizontal dimensions. Children were instructed to compare the stimuli with the reference number 5. Digits were presented in white on a black background. If the presented number was smaller than 5, the child had to press a predefined key on the left side of the keyboard, with the left hand. If the stimulus was higher than 5, then the key to be pressed was located at the right side and should be pressed with the right hand. The number 5 was never presented on the computer screen. Numerical distances between stimuli and the reference number (5) varied from 1 to 4, each numerical distance being presented the same number of times. Between trials, a fixation point of the same size and color of the stimuli was presented on the screen. The task comprised 80 experimental trials. The maximum stimulus presentation time was 4000 ms, and the intertrial interval was 700 ms.

#### Simple calculation

This task consisted of addition (27 items), subtraction (27 items), and multiplication (28 items) operations for individual applications, which were printed on separate sheets of paper. Children were instructed to answer as fast and as accurately as they could, with the time limit per block being 1 min. Arithmetic operations were organized at two levels of complexity and were presented to children in separated blocks: one consisted of simple arithmetic table facts and the other consisted of more complex facts. Simple additions were defined as those operations that had results of below 10 (i.e., 3 + 5), while complex additions had results between 11 and 17 (i.e., 9 + 5). Tie problems (i.e., 4 + 4) were not used for addition. Simple subtraction comprised problems in which the operands were below 10 (i.e., 9 − 6), while for complex subtractions, the first operand ranged from 11 to 17 (i.e., 16 − 9). No negative results were included in the subtraction problems. Simple multiplication consisted of operations that had results of below 25 and that had the number 5 as one of the operands (i.e., 2 × 7, 5 × 6), while for the complex multiplication, the result of the operands ranged from 24 to 72 (6 × 8). Tie problems were not used for multiplication. Reliability coefficients were high (Cronbach's α > 0.90).

#### Simple word problems

Twelve arithmetical word problems were presented to the child on a sheet of paper while the examiner read them aloud simultaneously to avoid a reading proficiency bias. There were six addition and six subtraction items, all of them with single-digit operands and results that ranged from 2 to 9 (i.e., “Annelise has 9 cents. She gives 3 to Pedro. How many cents does Annelise have now?”). The child had to solve the problems mentally and write the answer down in Arabic format as quickly as possible, and the examiner registered the time that was taken for each item. Cronbach's α of this task was 0.83.

#### Arabic number reading task

*Twenty-eight* Arabic numbers printed in a booklet were presented one at a time, to the children, who were instructed to read them aloud. The item set consists of numbers up to 4 digits (3 one-digit numbers, 9 two-digit numbers, 8 three-digit numbers, and 8 four-digit numbers). There were 12 numbers that could be lexically retrieved, 5 numbers that required three transcoding rules according to the ADAPT model (Barrouillet et al., 2004) to be correctly read, 6 numbers with four rules and 5 numbers with more than five rules. The internal consistency of the task is 0.90 (KR-20 formula) (Moura et al., 2013).

#### Arabic number writing task

Children were instructed to write the Arabic form of dictated numbers. This task is composed of 40 items, with up to 4 digits (3 one-digit numbers, 9 two-digit numbers, 10 three-digit numbers, and 18 four-digit numbers). The one- and two-digit numbers were classified as “lexical items” (12 items), and the other 28 items were subdivided according to the number of transcoding rules based on the ADAPT model (Barrouillet et al., 2004; Camos, 2008). There were six numbers that require 3 rules, nine numbers that require 4 rules, six numbers with 5 rules, five numbers with 6 rules, and two numbers with 7 rules. The internal consistency of this task is 0.96 (KR-20 formula) (Moura et al., 2013).

## Results

### General cognitive assessment

**Figure 2:**
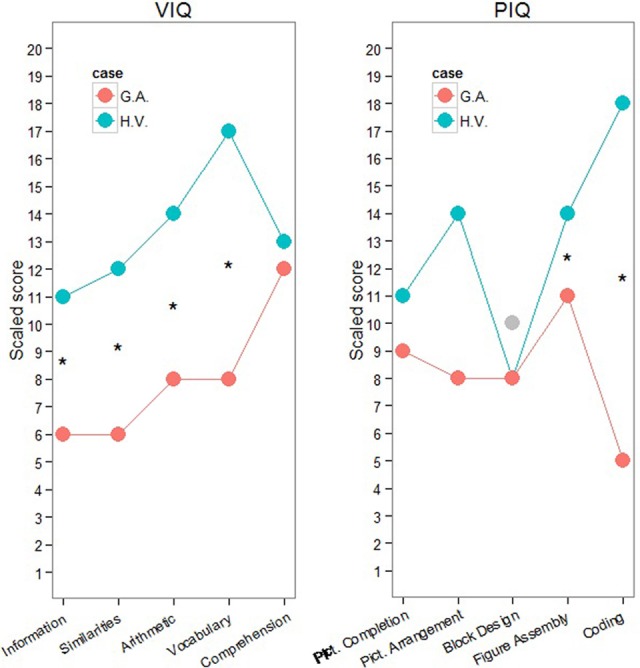
**H.V. and G. A. performances in WISC-III**. ^*^Marked statistical significance at the level *p* < 0.001. Note: as H.V.'s standardized Block Design score was below the mean in the first assessment, this subtest was repeated two years later (gray dot). The new standardized score in Block Design was equal to 10.

**Figure 3:**
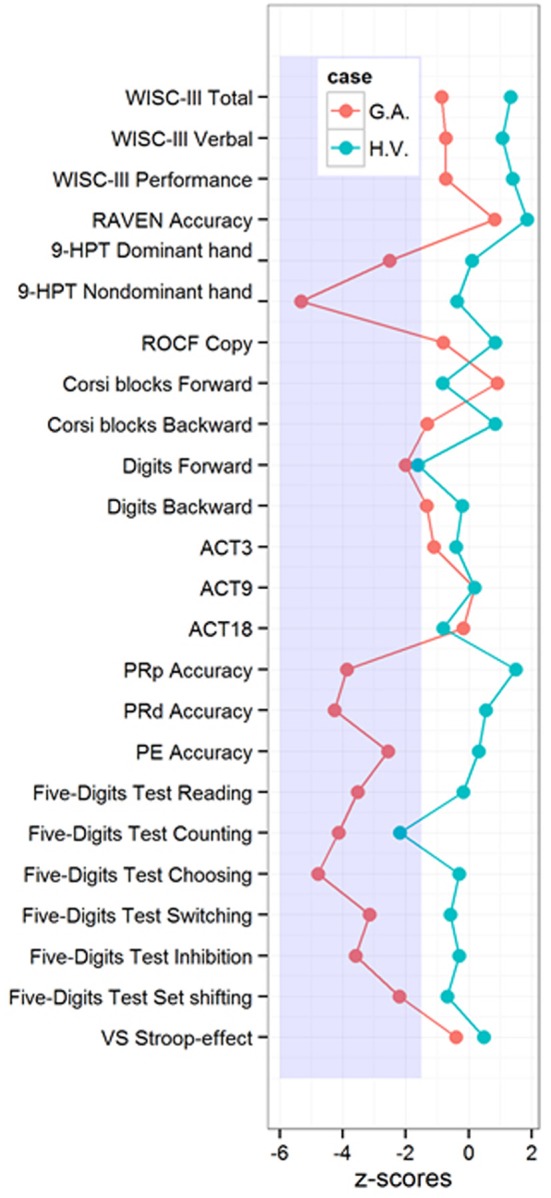
**H.V. and G. A. performances in intelligence, motor dexterity, visuospatial, short-term and working memory, executive functions, and phonological processing tasks**. 9-HPT, Nine Hole Peg Test; ROCF, Rey-Osterrieth Complex Figure; ACT, Auditory consonantal trigrams; PRp, Pseudoword Repetition; PRd, Pseudoword Reading; PE, Phoneme Elision; VS, Victoria Stroop. Clinical score < −1.5 *SD*.

Results of the CBCL reported by their respective mothers were in the normal range in all of the subscales (T-scores in the single subscales ranged from 37 to 45 in H.V. and from 36 to 54 in G.A. Scores above 70 are considered to be clinical). This finding indicates that both children have adequate levels of psychosocial functioning, according to their mothers. The results of the intelligence test are exhibited in Figure 2, while Figure 3 depicts comparative results in the two cases for the general neuropsychological assessment compared to norms from the original publications. H.V. shows a performance in the upper bound of normal intelligence (Raven's *PR* = 99, *FSIQ* = 120, *VIQ* = 116, and *PIQ* = 121), and G.A. shows average intelligence (Raven's *PR* = 75, *FSIQ* = 87, *VIQ* = 89, and *PIQ* = 89).

Statistical comparisons between both children in the subtests that measure the verbal and performance IQs (Huber, 1973; Willmes, 1985) reveal significantly higher scores for H.V. in the subtest Information (*Z* = 2.95; *p* = 0.016), Similarities (*Z* = 3.33; *p* = 0.004), Arithmetic (*Z* = 2.58; *p* = 0.05), Vocabulary (*Z* = 4.87; *p* = 0.00001), Figure Assembly (*Z* = 2.36; *p* = 0.01) and Coding (*Z* = 5.59, *p* = 0.000001). These results disclose a general pattern of higher scores in H.V. than in G.A. regarding tasks that demand more from verbal IQ but not as much regarding performance IQ.

Performance on the TDE (Brazilian School Achievement Test) was below the 25th percentile in both cases for Arithmetic. H.V.'s accuracy percentage was 29% (raw score = 11, grade mean = 16 grade, *SD* = 3.39) and G.A.'s was 36% (raw score = 14, grade mean = 16, grade *SD* = 3.39). The 25th percentile criterion is used as a lenient cut-off and is sensitive to math learning difficulties (Mazzocco, 2007; Landerl and Kölle, 2009; Landerl et al., 2009). Performance on the single word Reading and Spelling subtests of the TDE were normal for H.V. and below the 25th percentile for G.A. G.A. solved 14 out of the 35 items of the Spelling subtest correctly. In some items, he excluded phonemes (especially /r/, regardless of its mode or place of articulation), and in others, he confused phonemes that have similar sounds (such as v/f; m/n; b/d; and s/c). He clearly presented a phonological writing pattern, but he still lacks the mastery of the alphabetical principle. In the Reading subtest, G.A. could read 55% of the single words (raw score = 39, grade mean = 64.75, *SD* = 4.67), and his reading was extremely slow. He struggled at reading consonant clusters.

Regarding motor dexterity in the 9-HPT, H.V. did not present any major difficulties, whereas G.A.'s score was on the adopted clinical range, which means that he was significantly slower than would be expected for his age range according to Poole et al. (2005) (Figure 2). Both children presented right hand dominance (Lefèvre and Diament, 1982) as well as normal right-left orientation (Dellatolas et al., 1998) and finger gnosias (Dellatolas et al., 1998) (Figure 2). Neither of the children presented visuospatial constructional deficits.

On the phonological processing tasks, G.A. was significantly worse on all of the tests that were used, while H.V. presented typical scores. G.A. presented difficulties in storing and reproducing pseudowords as well as in reading them. In addition, he was not able to grasp the grapheme-phoneme correspondence principle that is needed to perform the phoneme elision task.

Both children presented difficulties in the phonological short-term memory task (forward digit span), but in both cases, scores in the phonological working memory tests (backward order of the Digit Span as well as the Auditory Consonantal Trigrams) fell into the expected ±1.5 *SD* range (Figure 2). This specific difficulty on the forward order of the Digit Span was mild, and it can be attributed to attentional lapses (Strauss et al., 2006). G.A. presented a better performance on the forward order of the Corsi Blocks compared to the backward, and H.V. showed the opposite pattern. However, both of their spans were in accordance to what would be expected for their age range. The performance of both children was in the typical range for the Victoria Stroop task. G.A.'s performance was in the clinical range for all of the subtests of the Five-digits test, those that involve more automatic processing (speeded digit reading and counting) as well as those that require executive functioning (inhibition and shifting). H.V. presented only a specific impairment that involved counting skills on the Five-digits Test, which will be discussed in more detail below^1^.

### Math cognitive assessment and computer tasks

Results of the computerized and math-cognitive tasks are shown in Tables 2, 3 for H.V. and in Tables 4, 5 for G.A. and their respective control groups.

**Table 2:** **Descriptive data and comparison between the control groups and H.V., in the alertness and number sense tasks**.

**Domain assessed**	**Task**	**Controls (*n* = 08)**		**H.V**.	**Modified *t*-test**	***p***	**Z-CC**	**Estimated % pop. below H.V**.
		**Mean**	***SD***					
Alertness	Simple manual reaction time	413.3	32.9	381.3	−0.92	0.39	−0.97	19
Number sense	**SYMBOLIC MAGNITUDE COMPARISON**							
	Response time^*^	771.6	154.8	1153.3	2.32	0.05	2.47	97
	Weber Fraction	0.27	0.17	0.12	−0.83	0.21	−0.88	22
	**NON-SYMBOLIC MAGNITUDE COMPARISON**							
	Response time^*^	1035.5	199.5	1003.2	−0.15	0.88	−0.16	44
	Weber fraction	0.29	0.06	0.42	2.24	0.04	2.17	4

ZCC: magnitude effect index calculated by the difference between the scores of the control group and the single case with a 95% CI (Crawford et al., 2010);

* *time in milliseconds*.

**Table 3:** **Descriptive data and comparisons between control groups and H.V. in the Simple calculation, Simple word problems, and Verbal-Arabic transcoding tasks (*df* = 1)**.

**Domain assessed**	**Task (total of items)**	**Controls (*n* = 08)**		**H.V**.	**X^2^**	***p***
		**Mean**	***SD***			
Simple calculation	**BASIC ARITHMETIC OPERATIONS**					
	Simple addition (12)	11.88	0.35	9	1.30	0.25
	Complex addition (15)	13.88	2.23	7	5.45	0.02
	Simple subtraction (12)	10.50	1.69	11	<0.01	1.00
	Complex subtraction (15)	8.50	3.25	4	1.68	0.19
	Simple multiplication (15)	13.13	2.64	4	11.18	0.00
	Complex multiplication (13)	6.25	3.45	0	5.81	0.02
Simple word problems	Math word problems (12)	10.50	2.35	12	0.18	0.67
Verbal-Arabic transcoding	Arabic number writing task (40)	38.00	3.22	40	0.51	0.47
	Arabic number reading task (28)	27.75	0.71	28	<0.01	1.00

**Table 4:** **Descriptive data and comparison between control groups and G.A. in the alertness and number sense tasks**.

**Domain assessed**	**Task (total of items)**	**Controls (*n* = 17)**		**G.A**.	**Modified *t*-test**	***p***	**Z-CC**	**Estimated % pop. below G.A**.
		**Mean**	***SD***					
Alertness	Simple manual reaction time^*^	423.8	82.3	447.9	0.29	0.39	0.29	39
Number sense	**SYMBOLIC MAGNITUDE COMPARISON**							
	Response time^*^	983.1	249.4	1344.9	1.41	0.09	1.45	9
	Weber fraction	0.21	0.14	0.78	3.96	<0.001	4.07	>99
	**NON-SYMBOLIC MAGNITUDE COMPARISON**							
	Response time^*^	1276.3	294.9	1038.2	−0.79	0.22	−0.81	39
	Weber fraction	0.28	0.10	0.21	−0.68	0.25	−0.70	25

ZCC: magnitude effect index calculated by the difference between the scores of control group and single-case with a 95% CI (Crawford et al., 2010);

* *time in milliseconds*.

**Table 5:** **Descriptive data and comparison between control groups and G.A. in the Simple calculation, Simple word problems, and Verbal-Arabic transcoding tasks (*df* = 1)**.

**Domain assessed**	**Task (total of items)**	**Controls (*n* = 17)**		**G.A**.	**X^2^**	***p***
		**Mean**	***SD***			
Simple calculation	**BASIC ARITHMETIC OPERATIONS**					
	Simple addition (12)	11.71	0.69	6	4.78	0.03
	Complex addition (15)	10.12	3.06	5	2.26	0.13
	Simple subtraction (12)	10.41	2.15	8	0.46	0.50
	Complex subtraction (15)	5.18	3.21	2	0.87	0.35
	Simple multiplication (15)	9.71	4.58	5	1.84	0.18
	Complex multiplication (13)	2.35	2.12	0	0.85	0.36
Simple word problems	Math word problems (12)	10.82	1.19	4	5.98	0.01
Verbal-Arabic transcoding	Arabic number writing task (40)	38.65	2.78	26	10.94	<0.001
	Arabic number reading task (28)	27.59	0.87	16	11.61	<0.001

In the simple reaction time task, H.V. did not show any impairment. In contrast, she responded faster than the average of her group. In the symbolic number task, the picture is different. Although H.V. was significantly slower than her control group, her response accuracy was slightly higher than that of controls in a type of speed-accuracy trade-off. Moreover, the performance of H.V. in the nonsymbolic task was markedly impaired in comparison with her control group. While the reaction times were comparable to the group average, the accuracy was very poor, especially for the more difficult numerical ratios. These deficits added to the picture that was formed by a speeded counting impairment in the Five-digits Test. The results of the number processing tasks suggest that there was a specific impairment in the number sense acuity in the presence of relatively spared numerical symbolic abilities.

H.V.'s performance was substantially impaired in complex addition and multiplication operations. Her performance was comparable to the control group in simple word problems (Table 3). H.V. can solve simple addition and subtraction operations as accurately as expected according to her age. In complex addition operations, H.V. presents more difficulties when compared to her control group. Interestingly, these difficulties could not be observed in complex subtraction tasks. Moreover, in comparison to controls, H.V. shows systematic difficulties when solving simple and complex multiplication operations, which can be interpreted as a more general deficit in fact retrieval. No deficits were observed in simple word problems with one-digit operands, the solution of which depends on text comprehension; these problems can be solved by counting procedures. She solved all of the problems correctly but took considerably more time to reach the correct results. Performance on number transcoding of three- and four-digit numerals was comparable to the control group (Table 3). These results are summarized in Table 3.

In the simple reaction time task, G.A. did not show any impairment but instead showed average performance (Table 4). In the symbolic number task, G.A. responded tendentially slower and much less accurately than his control group. In contrast, G.A. presented both average response latency and average accuracy in the nonsymbolic number comparison task. In the number processing tasks, G.A. experienced considerable difficulties in tasks that use the symbolic notation and verbal procedures, such as speeded counting, speeded digit reading, transcoding and symbolic magnitude comparison (up to nine). G.A.'s pattern of impairment in the math tasks contrasts with that of H.V. Difficulties in the symbolic number processing tasks in G.A. are at odds with a normal Weber fraction.

G.A.'s difficulties with the symbolic processing were also corroborated by his lower performance in the transcoding tasks. In the number writing task, G.A. committed 14/40 errors. G.A. presented three lexical errors (all of them were related to phonological resemblance between the trial and the number written by him) and eleven syntactic ones (seven being related to adding internal zeros and four to deleting a digit). Fifty-two percent of his control group did not commit any error. From the eight children who did, one committed eleven errors, one presented five errors, one committed two errors and the other five children made only one single mistake.

The lexical mistakes by G.A. clearly have a phonological bias. In Portuguese, the numbers “three” and “six” sound very similar (“*três*” and “*seis*,” respectively), as well as “seven hundred” and “six hundred” (“*setecentos*” and “*seiscentos*”). Moreover, the syntactic errors of G.A. always involved the addition principle (overwriting rule, Power and Dal Martello, 1990; Moura et al., 2013). G.A. wrote the number 643 as 646 and 4701 as 400601. His performance on the number reading test also corroborates his difficulties with place value understanding. He read the number “2000” as “*two hundred*” and “1013” as “*one hundred thirteen*.” On two items, he decomposed the numbers: 567 was read as *“five and sixty seven*” and 5962 as “*fifty nine and sixty two*.” Nevertheless, the mistakes made by G.A. cannot be easily attributed to a lack of knowledge of the rules of additivity in number transcoding. G.A. was able to transcode correctly five out of eleven complex numbers with syntactical zeros (e.g., “109,” “902,” “1060,” “1002,” and “7013”) but failed to transcode numbers of comparable complexity (“101” ≥ 11, “1015” ≥ 10015, “2609” ≥ 20069, “4701” ≥ 40601, “1107” ≥ 2067, and “7105” ≥ 715). Therefore, the poor transcoding performance of G.A. is compatible with deficits in phonological representations combined with problems with concentration and monitoring capacity. Evidence for a deficit in knowledge about the structure of the Portuguese verbal number system was not obtained.

Difficulties with simple word problems were more severe. G.A. did not show any impairment in solving addition, subtraction and multiplication problems when compared to controls, except for a single result that indicated lower performance while solving simple addition tasks (Table 5). This pattern is consistent with the mother's report that G.A. acquired the arithmetic facts after struggling with them for a while. However, the verbal nature of G.A.'s difficulties becomes explicit again, when considering his attainment of simple word problems. From 12 problems, G.A. solved only 4 correctly, responding sometimes with absurd values, which suggested that he was guessing. His performance on word problems was almost six standard scores below that of the controls. In summary, the results of the math cognitive investigation suggest that G.A.'s difficulties in learning math can be attributable to his comorbid reading learning disability.

## Discussion

In the present study, we selected two cases that had relatively specific impairment patterns from an outpatient clinic for mathematical learning disorders and conducted a detailed neuropsychological and cognitive assessment with the aim of characterizing possible endophenotypes. Specificity of the impairments is corroborated by the fact that both children were of average or above average intelligence and did not present impairments in visuospatial and visuoconstructional processing, as assessed by the Rey figure copy and Block Design subtest of the WISC. In the following, we will discuss the extent to which the neuropsychological profile of H.V. and G.A. fitted specific endophenotypes, as predicted in the literature.

### H.V.

Difficulties in H.V. are specific, severe and persistent and were restricted to an inaccurate number sense and to the acquisition of arithmetic facts, which reflected mostly on multiplication operations. H.V. is curious and motivated to learn, except for mathematics. H.V. has difficulties in memorizing even the simplest arithmetic facts, but she is highly skilled in finger counting. The single abnormally lower score observed in the general neuropsychological assessment was in the forward version of the digit span. An excellent performance was observed in reading-related phonological processing tasks, such as pseudoword repetition, pseudoword reading and phonemic ellison. No abnormalities were observed in executive function tasks.

One might wonder why the performance of H.V. in the subtest “counting” of the Five-digits Test of executive functions was so low and discrepant from her general level of performance on this test. The subtest counting is a speeded task in which one has to count how many stars are printed on a series of cards that display sets of one up to five stimuli. The difficulties with the speeded counting of stars presented by H.V. reflect much more a deficit in the apprehension of nonsymbolic magnitude information under time constraints. This pattern contrasts with her resourceful use of strategies to compensate for her difficulties in other tasks that do not require nonsymbolic number processing. One of her favorite compensatory strategies for solving even the simplest arithmetic problems is finger counting. Once sufficient time is allowed, H.V. can find the correct response by finger counting. Her difficulties are accentuated in speeded tasks that require automatic retrieval.

The deficits in fact retrieval that are presented by H.V. cannot be attributed to a reduced capacity of verbal working memory or phonological awareness because H.V. shows high levels of competence in these two cognitive functions. However, the deficits in the numerical and arithmetic abilities of H.V. are compatible with generally imprecise or poor numerical representations: on the one hand, the deficits of H.V. in multiplication tasks suggest impairment in the retrieval of appropriate information from memory. On the other hand, the high value of the Weber fraction observed in nonsymbolic magnitude comparison suggests a very inaccurate ANS.

In contrast to our expectations, the profile of H.V. does not fit a typical endophenotype that is characterized by a number sense deficit (Wilson and Dehaene, 2007; Noël and Rousselle, 2011). Although H.V. presents low acuity in nonsymbolic magnitude comparison, this deficit is not present in the symbolic version of the task. More importantly, a substantial deficit in arithmetic operations—particularly in subtraction—was not observed. In contrast, H.V. presented some deficit in complex addition operations, but no sign of a deficit was observed in simple or complex subtraction operations. Moreover, a substantial deficit in multiplication operations (simple as well as complex) cannot be accounted for by a deficit in the number sense alone, but suggests the presence of difficulties for automatizing the retrieval of multiplication facts.

### G.A.

G.A. presented persistent but milder difficulties in learning math in the context of developmental dyslexia with severe associated phonemic processing deficits. In the case of G.A., math learning impairments were observed in transcoding operations as well as in very simple one-digit word problems. G.A. presented deficits in all phonological processing tasks: digit span, pseudoword repetition and reading as well as in phoneme elision. Although his intelligence is normal, difficulties were also observed in motor dexterity and in all subtests of the five-digits procedure, both those tapping automatic (speeded counting, speeded digit reading) and those assessing controlled processing (inhibition, set shifting). Moreover, a borderline performance was also observed in the forward and backward Digit and backward Corsi spans.

G.A. showed a less pronounced deficit in numerical and arithmetical abilities than H.V. The acuity of his representation of magnitude was comparable to controls, as measured by the nonsymbolic magnitude comparison task. In contrast, in the symbolic magnitude comparison task, G.A. committed many more errors and was marginally slower than his control group. Although G.A. presented lower levels of performance than controls in the simple addition operations, no other difference was observed in simple or complex addition, subtraction or multiplication operations. This pattern indicates that G.A. can retrieve from memory the correct responses to simple operations and employ the correct procedures to execute more complex addition and subtraction operations. However, in comparison to the controls, G.A. was much less successful when solving word problems. G.A. also presented substantially more difficulties in transcoding tasks in comparison to his peers, especially regarding phonological representations, concentration and monitoring capacity.

The profile of G.A. fits only partially a typical endophenotype that is characterized by a verbal and symbolic deficit. Although G.A. presents low acuity in symbolic magnitude comparison, simple word problems and impaired performance in transcoding tasks, this deficit does not extend to the retrieval of multiplication facts. It is still a matter of debate to what extent multiplication facts are stored in a typical verbal format (Varley et al., 2005; Benn et al., 2012). However, deficits in verbal numerical information processing have, very often, been associated with deficits in fact retrieval (De Smedt and Boets, 2010; De Smedt et al., 2010).

G.A. also presents severe problems with motor dexterity, which are assessed with the 9-HPT, which deserve consideration. Sensorimotor impairments are a frequent concomitant of specific learning disorders observed both in dyslexia (White et al., 2006a,b) and in dyscalculia (Costa et al., 2011; Lonnemann et al., 2011). Minor sensorimotor dysfunction was observed in 87% of dyslexic children with an IQ higher than 85 (Punt et al., 2010). In this context, they are not interpreted as a causal mechanism that is implicated in learning difficulties, but as markers or co-localizers of brain insult (Denckla, 1997, 2003; Batstra et al., 2003). Whatever the cause of G.A.'s present learning difficulties, it also impaired his neurological functions in a more widespread manner, as shown by the relatively severe reduction in motor dexterity. Because the motor difficulties were comparable in both hands, no inferences can be made regarding lateralization of the underlying pathological process, other than the left-hemisphere dysfunction that is connected to developmental dyslexia.

In our view, the sensorimotor deficits could be responsible for his deficits in other tasks as well. G.A.s performance in both the Block Design subtest and Rey's Figure copy were situated from 0.7 to 1 standard deviations below the mean, which suits his WISC-FSIQ of 90. Moreover, a qualitative assessment of G.A.'s performance in the Block Design subtest and Rey's Figure copy indicate that his relative difficulties originate from the motor dexterity and executive components that are mobilized to solve these tasks and do not reflect impairments in apprehension or reproduction of visuospatial configurations. Further corroboration of these findings comes from the Raven. There, G.A. reached a score that was higher than average. In our view, such a level of performance on the Raven cannot be reached when simple visuospatial processing is impaired.

The difference between G.A.'s scores on the WISC and Raven can be attributed to an interaction between test and individual characteristics. Compared to the Raven, the WISC-III imposes greater demands on verbal and scholastic abilities. Performance on several WISC tasks is also time constrained. We believe that G.A.'s relatively lower performance on the WISC can be explained by his reading and academic difficulties as well as by impairment in motor dexterity and processing speed. This pattern is especially salient on the Coding subtest, which is the test that presents the worst performance. Difficulties with the Coding subtest can also be related to G.A.'s impairment with respect to the symbolic transcoding tasks (Strauss et al., 2006).

### Specific deficits in automatic vs. controlled numerical processing?

Comparisons of the endophenotypes as predicted by the current literature (Wilson and Dehaene, 2007; Noël and Rousselle, 2011) and the individual cases of H.V. and G.A. yield apparently frustrating results because the performance of H.V. and G.A. on the arithmetic tests partly contradicts the general expectation of more or fewer specific deficits in the number sense and verbal numerical representations, respectively. One possible interpretation of these results is that paradigmatic cases that regard specific endophenotypes can be very difficult to find. Although the initial assessment of H.V. and G.A. suggested number sense and verbal deficits, a more detailed examination revealed, in both cases, a less precise picture. Similar difficulties encountered by other authors (e. g., Tressoldi et al., 2007), suggest that only a small proportion of all of the cases of mathematics difficulties can reveal more pure forms of endophenotypes. This finding raises the question about the proportion of cases of mathematics difficulties that can actually be assigned with confidence to one or another subtype of this disorder. If it is low, then the general approach of endophenotypes might prove to be ineffective. Although our case design does not allow a direct investigation of this question, in this section, we will discuss one possible reason why endophenotypes can be indeed valuable in the investigation of mathematics difficulties.

One could propose that the severe deficits of H.V. solving multiplication problems while simultaneously being capable of solving complex subtraction problems are a result of compensatory strategies, such as finger counting. Finger counting could be more effective for subtraction than for multiplication operations because the multiplication operations usually have much higher numbers as the answers, which are much more difficult to reach by counting. Assuming that this reason explains H.V.'s performance, the discrepancy between her performance and the typical results that are expected according to the number sense endophenotype should be due to relatively trivial differences between prototypical profiles and individual cases, without more profound consequences for the refinement of the theoretical framework of mathematical learning disorders.

The same conclusion can be reached when analysing the discrepancy between G.A.'s performance and a verbal numerical endophenotype. Deficits in calculations should be expected, especially when the problems are more complex, rely more strongly on a verbal code, and the ability to use verbal number representations is as limited as in the case of G.A. However, this expectation was not confirmed by the results. Once more, one can attribute the discrepancy between the observed performance and typical endophenotypes to some individual compensatory resource, which is always plausible in individual cases and is frequently reported in clinical observations (Temple and Clahsen, 2002; Thomas and Karmiloff-Smith, 2002).

Moreover, the cognitive-neuropsychological approach to developmental disorders has been criticized on the grounds of the dynamics of the developing brain (Thomas and Karmiloff-Smith, 2002). Early acquired lesions or genetic dysfunctions can induce varying degrees of reorganization in the cognitive relevant brain processes. In exceptional cases, clear-cut structural-functional correlations, which are similar to the ones encountered in adults, are observed in cases of dysfunction in the developing brain (e.g., Temple, 1989, 1991; Sullivan et al., 1996; Ta'ir et al., 1997). In most cases of early acquired or genetic disorders, clinical-anatomical correlations are attenuated by several neuroplastic and compensatory processes.

Interestingly, there is an aspect of the performance of both H.V. and G.A. that could account for the patterns of the results observed in the respective cases without resorting to weak accounts that are based on typicality. The pattern of performance presented by H.V. reveals deficits in different numerical representations, which usually can be operated in an automatic or effortless fashion. The definition of the ANS, for example, involves an intuition for magnitudes and the capacity to activate it in a very automatic way (Dehaene, 1992; Verguts and Fias, 2008; Hyde, 2011). Moreover, the capacity to retrieve arithmetic facts appears to be a very automatic process as well (Domahs and Delazer, 2005; Zamarian et al., 2009). Such a specific deficit in the automatic access to information regarding, on the one hand, the ANS, and on the other hand, multiplication facts can account for the apparently discrepant deficits that are presented by H.V. A core deficit in the number sense alone cannot account for H.V.'s isolated deficits in multiplication but lack of deficit in subtraction operations of comparable difficulty.

On the other hand, the patterns of deficits presented by G.A. are suggestive of difficulties with a more executive and effortful processing of numerical representations as well as mit some aspects of effortless processing. The spared performance of G.A. in all arithmetic operations is compatible with this view because the problems employed in the present study never had operands that were larger than two-digits, with which G.A. has had sufficient experience in the past. In contrast, the transcoding task employed much larger numbers. This more complex part of the verbal numerical system is learned for the first time exactly in the grade that G.A. was attending during his assessment. This finding is suggestive that G.A. still needs substantial executive resources to employ correctly the transformation rules that are necessary to transcode those numbers (Barrouillet et al., 2004; Camos, 2008; Moura et al., 2013). More detailed analysis of G.A.'s poor transcoding performance reveals no evidence for a deficit in knowledge about the structure of the Portuguese verbal number system. In contrast, G.A.'s error pattern is indicative of severe problems with phonological representations, concentration and monitoring capacity. Accordingly, orally presented word problems can also be more challenging for G.A. because a good capacity in verbal working memory is necessary to select relevant information from these problems and then operate with them until the correct result is obtained.

Support for this interpretation of H.V. and G.A. endophenotypes comes also from the analysis of the Five-digits Test results (see Figure 2). The Five-digits Test is well-suited to perform this comparison because the stimuli and task context are preserved, while the cognitive demands in terms of automatic and controlled processing vary (see also van der Sluis et al., 2004). On the one hand, H.V. presents difficulties with speeded counting but does not present difficulties with the inhibition- and shifting-demanding tasks. G.A., on the other hand, encounters difficulties in all aspects of the task, which requires both automatic and controlled processing. G.A.'s pattern of performance in the Five-digits Test is similar to the pattern observed by van der Sluis et al. (2004) on an equivalent numerical task in children with math learning difficulties and both math and reading learning difficulties. Interactions between processing speed and working memory impairments have been observed in several studies of both typically developing children (Berg, 2008) and children with math learning disability (Bull and Johnston, 1997). Moreover, disorders of automatization and procedural learning have also been implicated in learning disabilities of both reading (Menghini et al., 2006) and arithmetic (Lonnemann et al., 2011). Our results suggest that, in some cases, difficulties can be more related to the automatic or effortless processing, with possible compensation through more controlled strategies (H.V.), while in other cases, difficulties could be mixed or impairing more heavily controlled forms of processing (G.A.).

Overall, these results suggest that the search for endophenotypes could be more complex than originally expected, but not useless. In contrast, endophenotypes could be the only way to disclose more precise details on the nature and extension of mathematics difficulties. The current models of mathematics difficulties (e.g., Rubinsten and Henik, 2009) treat the different subtypes of math difficulties as members of a class of disorders that have different natures, which are nevertheless at more or less the same hierarchical level of organization of the cognitive system. This model has been proven to be useful but requires better specification.

One might consider the role of good executive functioning resources as a compensatory mechanism in developmental disorders. Johnson (2012) has proposed a role for executive functions in compensating for developmental neurogenetic impairments. According to this view, impairments in more basic and modularly organized aspects of information processing, such as phonological processing and number sense, can be compensated for if they are not sufficiently severe or if the individual has good executive functioning resources. The expression of symptoms that lead to diagnosis would occur in cases in which specific processing deficits are severe or when executive functioning resources are not sufficient to meet the environmental demands. The pattern of deficits presented by H.V. and G.A. are in line with these arguments. While H.V. was able to mobilize resources from executive functions and compensate for many of her deficits in number processing, the same could not be observed in the case of G.A.

Moreover, H.V.'s case also suggests that, in addition to executive functions, a more basic level of task automatization should be considered to be a bridge between domain-specific and domain-general cognitive impairments that contribute to math learning difficulties. This topic has received less consideration in the literature (however, see van der Sluis et al., 2004; Chan and Ho, 2010).

Automatic and controlled processing are two dimensions of cognitive abilities that interact with domain-general and -specific factors, and the neurobiological basis of these processes should also be examined in more detail. Contemporary models of skill learning and automatization assume that, in the initial steps of learning, higher demands on processing are imposed over the fronto-parietal circuits that underlie cognitive control (Schneider and Chein, 2003). With practice, the typical focus of activity is shifted from anterior cortical regions to posterior ones and to the striatum. Another assumption is that this anterior-to-posterior shift in activity is domain-general because this circumstance has been observed with several motor and cognitive tasks. The extant literature largely supports these assertions (Patel et al., 2012). Similar observations have been made in the domain of numerical cognition. Interference effects in a number-size interference task are related to activation in frontal areas, while the distance effect is associated with activation in parietal areas, including the intraparietal sulcus (Kaufmann et al., 2005). Learning arithmetic facts is followed by a shift of the activation focus from frontal and intraparietal areas to the left angular gyrus (Zamarian et al., 2009). Developmentally, children usually activate more widespread areas during mental calculations, including frontal regions (Kawashima et al., 2004; Rivera et al., 2005). In adults, the focus of activity is more concentrated on posterior areas (Kaufmann et al., 2008, 2011; Klein et al., 2009).

Available evidence on the neurocognitive underpinnings of skill learning and automatization allow us to tentatively predict structural-anatomical correlations of automatic and controlled processing impairments in math learning difficulties. Numerical-specific automatic processing deficits, such as the deficits presented by H.V., should be related to impairments in parietal areas, including connections to the intraparietal sulcus. A broader pattern of dysfunction, encompassing the frontal areas, should be observed in cases such as G.A., in whom controlled processing is also impaired. Obviously, math learning difficulties that are associated with dyslexia also imply malfunctioning of perisylvian areas.

Results of the present paper have important implications for future research. The first implication is the need to include both domain-specific and domain-general measures to fully describe the range of manifestations and impairments in math learning difficulties (Cowan and Powell, 2013). Moreover, the neuropsychological test batteries that are used to assess math learning should fairly measure both automatic or effortless processing and effortful or controlled processing. Because comorbidity with ADHD can explain impairments in working memory and executive functions, ADHD symptoms should also necessarily be controlled. Otherwise, it is not possible to draw straightforward conclusions on how working memory/executive functions determine more general performance difficulties compared to numerical-specific deficits (Willburger et al., 2008). Tasks that assess working memory and executive functions should also be presented in two formats, using non-numerical and numerical stimuli (Raghubar et al., 2010). Another important implication is the need to assess more automatized number processing, such as RAN. Finally, we believe that the present study contributes to underline the importance of single-case research in clarifying the role of distinct endophenotypes in dyscalculia research.

In the present study, we demonstrated that automatic and controlled information processing is one valid and necessary axis of investigation when characterizing the multitude of cognitive deficits that are associated with math difficulties, which can conciliate apparent discrepancies between individual and typical endophenotypes with respect to math difficulties. This approach constitutes a more general level of description of cognitive deficits as that originally adopted by other authors in previous studies. In summary, phenotypic manifestations of learning disabilities are compounded by impairments in both specific and general information processing mechanisms. Math-specific factors, such as number sense, and math-nonspecific cognitive factors, such as phonological processing, interact with general aspects of information processing, such as controlled processing and automatization. Math-specific and more general information processing deficits and automatic and controlled information processing deficits therefore represent orthogonal but interacting dimensions of the same disorder. In this sense, symptoms would be apparent when general or specific compensatory mechanisms are overloaded or not sufficient to meet the environmental demands in cases of more specific impairments. Impairments in restricted, specific domains could explain the unique difficulties, while impairment in more general mechanisms could be related to the degree and form of phenotypic expression via compensatory mechanisms.

### Conflict of interest statement

The authors declare that the research was conducted in the absence of any commercial or financial relationships that could be construed as a potential conflict of interest.

## Footnotes

^1^After the neuropsychological assessment, both children initiated interventions based on cognitive-behavioral techniques to reduce math-anxiety symptoms and also to improve self-efficacy. Strategies such as problem-solving, self-monitoring, and self-reinforcement are coupled with errorless learning, allowing the kids to have experiences of academic success. Simultaneously, we also use instructional and training interventions that focused on number processing and arithmetic components that were considered to be impaired in each child. H.V. and G.A. have been participating in individual intervention programs for 4 semesters, 2 h a week. Their families also received counseling by means of a behavioral training program for one semester, once a week. During this time, H.V. has not obtained improvements in her number sense acuity, but she considerably improved in solving addition and multiplication problems. She has not automatized fact retrieval yet. H.V. does her homework with a pocket calculator. Initially, G.A. received training in text processing abilities and improved in arithmetical word problem solving. He also obtained substantial improvement in his transcoding abilities.
